# Joint effects of traffic-related air pollution and hypertensive disorders of pregnancy on maternal postpartum depressive and anxiety symptoms

**DOI:** 10.1038/s41370-024-00692-9

**Published:** 2024-05-31

**Authors:** Yuhong Hu, Thomas Chavez, Sandrah P. Eckel, Tingyu Yang, Xinci Chen, Mario Vigil, Nathan Pavlovic, Fred Lurmann, Deborah Lerner, Nathana Lurvey, Brendan Grubbs, Laila Al-Marayati, Claudia Toledo-Corral, Jill Johnston, Genevieve F. Dunton, Shohreh F. Farzan, Rima Habre, Carrie Breton, Theresa M. Bastain

**Affiliations:** 1 Department of Population and Public Health Sciences, Keck School of Medicine, University of Southern California, Los Angeles, CA USA; 2 Sonoma Technology Inc., Petaluma, CA USA; 3 Eisner Health, Los Angeles, CA USA; 4 Department of Obstetrics and Gynecology, Keck School of Medicine, University of Southern California, Los Angeles, CA USA; 5 Department of Health Sciences, California State University, Northridge, Northridge, CA USA

**Keywords:** Traffic-related air pollution, Postpartum mental health, Postpartum anxiety, Postpartum depression

## Abstract

**Background:**

Ambient air pollution has been linked to postpartum depression. However, few studies have investigated the effects of traffic-related NO_x_ on postpartum depression and whether any pregnancy-related factors might increase susceptibility.

**Objectives:**

To evaluate the association between traffic-related NO_x_ and postpartum depressive and anxiety symptoms, and effect modification by pregnancy-related hypertension.

**Methods:**

This study included 453 predominantly low-income Hispanic/Latina women in the MADRES cohort. Daily traffic-related NO_x_ concentrations by road class were estimated using the California LINE-source dispersion model (CALINE4) at participants’ residential locations and averaged across pregnancy. Postpartum depressive and anxiety symptoms were evaluated by a validated questionnaire (Postpartum Distress Measure, PDM) at 1, 3, 6 and 12 months postpartum. Multivariate linear regressions were performed to estimate the associations at each timepoint. Interaction terms were added to the linear models to assess effect modification by hypertensive disorders of pregnancy (HDPs). Repeated measurement analyses were conducted by using mixed effect models.

**Results:**

We found prenatal traffic-related NO_x_ was associated with increased PDM scores. Specifically, mothers exposed to an IQR (0.22 ppb) increase in NO_x_ from major roads had 3.78% (95% CI: 0.53–7.14%) and 5.27% (95% CI: 0.33–10.45%) significantly higher 3-month and 12-month PDM scores, respectively. Similarly, in repeated measurement analyses, higher NO_x_ from major roads was associated with 3.06% (95% CI: 0.43–5.76%) significantly higher PDM scores across the first year postpartum. Effect modification by HDPs was observed: higher freeway/highway and total NO_x_ among mothers with HDPs were associated with significantly higher PDM scores at 12 months postpartum compared to those without HDPs.

**Impact:**

This study shows that prenatal traffic-related air pollution was associated with postpartum depressive and anxiety symptoms. The study also found novel evidence of greater susceptibility among women with HDPs, which advances the understanding of the relationships between air pollution, maternal cardiometabolic health during pregnancy and postpartum mental health. Our study has potential implications for clinical intervention to mitigate the effects of traffic-related pollution on postpartum mental health disorders. The findings can also offer valuable insights into urban planning strategies concerning the implementation of emission control measures and the creation of green spaces.

## Introduction

Depression and anxiety, the most common mental health disorders across the globe, cost the global economy approximately 1 trillion US dollars each year in lost productivity [1–3]. It is estimated that 10–20% of U.S. postpartum women experience depressive or anxiety disorders [4, 5]. The symptom profile of anxiety includes excessive concern or worry that cannot be controlled, and intrusive thoughts, impulses, or behaviors, which occurs at a higher rate during the postpartum period than at other times [6]. Postpartum depression (PPD) is characterized by sad mood, restlessness and/or agitation, and impaired concentration, and is often accompanied by postpartum anxiety [4, 7]. Postpartum depression and anxiety have been linked with adverse outcomes among mothers and infants; however, they remain understudied.

Notably, Hispanic women in the US have unique risk factors for depression and other mental health disorders relative to non-Hispanic women including overcoming stress associated with acculturation as well as lower awareness and utilization of mental health care services [8–11]. Thus, it is of urgent public health priority to identify modifiable risk factors for depression to protect susceptible subgroups.

Epidemiological studies have suggested that long-term exposure to air pollution has been associated with depressive and anxiety symptoms among adults [12–15]. Animal studies have also shown that ambient particles can induce anxiety-like and depressive-like responses in mice [16, 17]. Studies further observe that airborne particulate matter may increase neuroinflammation and oxidative stress in the brain and may play a role in central nervous system (CNS) structural and functional changes associated with mental disorders [18–21].

Evidence is accumulating that prenatal exposure to air pollution is associated with adverse mental health disorders in the postpartum period. Pregnancy may be a particularly vulnerable window of air pollution exposure for maternal health effects, given the increased ventilation rate needed to support the developing fetus’ higher oxygen demands and the reduced capacity for oxygen binding [22]. Recent studies from our group and others have reported an association between ambient air pollution exposure during pregnancy and increased depressive symptoms in the postpartum period [5, 23–25].

Despite evidence that regional ambient air pollution affects postpartum depression, the association between local vehicle emissions and postpartum depressive and anxiety symptoms remains understudied. The residential ambient exposures used in previous studies of regional pollution cannot accurately estimate concentrations of the dynamic mixture of pollutants found in fresh vehicle emissions from local traffic, as their estimates encompass both the near-roadway and urban background concentrations [26]. Additionally, these ambient exposures estimated in larger scale did not differentiate the contributions of near-roadway sources from different road classes: (1) freeways and highways with the highest traffic volume but longest distance to people’s residence; (2) minor roads with the lowest traffic volume but shortest distance to people’s residence; (3) major roads with moderate traffic volume and distance to people’s residence.

Furthermore, a growing body of literature has indicated increased susceptibility to postpartum depression and anxiety in mothers with higher psychosocial and biological stress during pregnancy [27]. Additionally, the proinflammatory state observed in the late stages of pregnancy and the early postpartum period is also claimed to be relevant to PPD, given that elevated inflammation resulting from psychological or biological stressors is strongly linked to depressive symptoms in nonpuerperal depression in both animal and human studies [28–31]. Hypertensive disorders of pregnancy (HDP) are associated with elevated levels of inflammatory responses [32, 33], and epidemiological studies have reported that women with HDPs are more likely to develop postpartum depression and anxiety [34–36]. Studies have shown that inflammatory responses in gestational hypertension may be linked to oxidative stress and endothelial dysfunction [37]. Since the effect of air pollution on postpartum mental disorders has been suggested to be inflammation- and oxidative stress-mediated [18–21], there is biological plausibility that shared mechanisms underlie HDPs and postpartum depression and anxiety. Despite a growing number of studies showing that postpartum mental disorders are linked with higher levels of air pollution, less is known about the joint effects of pregnancy complications such as HDP and air pollution on postpartum anxiety and depression.

To date, there is a paucity of data regarding the association between traffic-related air pollution (TRAP) and postpartum depressive and anxiety symptoms in populations with lower socioeconomic status, and the putative role of pregnancy complications in this potential association remains elusive. To address these research gaps, we conducted a prospective study in a cohort of predominately low-income Hispanic/Latina pregnant women with high exposures to environmental pollutants. We investigated both time-specific association and association based on repeated measurement analyses between traffic-related NO_x_ and postpartum depression and anxiety across the first year of the postpartum period. Furthermore, we evaluated whether this association differed by the presence of HDP.

## Subjects and methods

### Study population and study design

The Maternal and Developmental Risks from Environmental and Social Stressors (MADRES) study is a prospective pregnancy cohort that primarily includes low-income, Hispanic/Latina women from Los Angeles, CA, who were recruited before 30 weeks of gestation at three community health clinics beginning in 2015. A detailed account of the study population and protocol can be found in a previous publication [38]. To be eligible for the study, participants had to be fluent speakers of either English or Spanish and over 18 years old. Women who were HIV positive, incarcerated, had multiple gestations, or had mental, physical, or cognitive disabilities that prevented informed consent were excluded from the study. Maternal written informed consent was obtained at the time of recruitment. Participants were compensated for their time completing study procedures. The Institutional Review Board at the University of Southern California approved all aspects of this study.

The participants included in this analysis were recruited from November 2015 through August 2022. Figure S1 shows the consort diagram for the study population included in this analysis. A total of 691 mothers delivered live births and reached at least 60 days postpartum by the cutoff day. Among these mothers, 526 participants completed the outcome assessment at least one time in the 1-year postpartum period and were not currently pregnant at time of assessment. After excluding participants with missing exposure and key covariate information, 453 mothers were included in the final analytical sample for this study.

### Exposure assessment

California LINE-source dispersion model (CALINE4) [39] was used to estimate prenatal traffic-related NO_x_ concentrations by road type at maternal residential locations. Traffic-related NO_x_ for each road class is a surrogate for the complex mixture of gases and particles emitted by motor vehicles and is also known as TRAP. Detailed data on roadway geometry, temporally and spatially resolved traffic volumes and speeds, vehicle fleet composition, hourly wind speed and direction, and atmospheric stability were used in CALINE4 model to estimate the concentrations from vehicle emissions downwind of roadways [40–42]. Vehicle emissions for the on-road fleet were estimated using the EMFAC 2017 emissions model [43]. CALINE4 NO_x_ estimates from all roads (total NO_x_) were built up from the contributions of three road classes: NO_x_ emitted from freeways and highways, NO_x_ emitted from major roads, and NO_x_ emitted from minor roads [41]. The distribution of the distance from participants’ residential addresses to roads of different classes is shown in Table S1, which is similar to what our group has previously found in the Southern California Children’s Health Study (CHS) [44]. Prenatal traffic-related NO_x_ exposures (freeways and highways/major roads/minor roads/total) were averaged across pregnancy. Trimester-specific associations were not reported due to the high temporal correlation between each trimester across pregnancy (Fig. S2).

### Outcome ascertainment

At approximately 1, 3, 6, and 12 months postpartum, we used a 9-item version of the Postpartum Distress Measure (PDM) to assess postpartum distress, including depressed mood and anxiety symptoms [45]. The PDM was developed to identify psychological distress, depressive, anxiety and obsessive-compulsive symptoms during the postpartum period. Reliability and validity of the PDM scale have been assessed and confirmed by previous studies [45]. Responses were recorded on a 0 to 3 Likert-type scale, and the total score was calculated by summing the responses to each item. Scores range from 0 to 27, with higher scores indicating greater symptoms of postpartum distress. Sample items include “I have recurring thoughts about my baby getting sick or having some kind of problem,” and “I check on my baby multiple times throughout the night.” The trajectory of PDM scores of each participant after childbirth is depicted in Fig. S3.

### Covariates and effect modifiers

We considered a wide range of potential confounders identified a priori using Directed Acyclic Graphs (DAGs, See Fig. S4) that could be associated with both exposure and outcome. The covariates included demographic and socioeconomic information (maternal age, race, education), and physiological characteristics (pre-pregnancy body mass index (BMI), parity). Interviewer-administered questionnaires in either English or Spanish were used to collect demographic information at study entry. Birth outcomes and related information, and depression history were abstracted from medical records (EMR). Maternal pre-pregnancy BMI was calculated using self-reported pre-pregnancy weight and measured height at a prenatal study visit. We also adjusted for the season of childbirth and calendar year of childbirth to account for temporal and seasonal variation in both exposure and outcome.

We assessed HDP as an effect modifier as it is an important pregnancy complication. HDPs usually occur after 20 weeks of gestation and include gestational hypertension, preeclampsia, and eclampsia. All preeclampsia and eclampsia cases and most gestational hypertension cases were ascertained using physician diagnosis abstracted from maternal EMR. Additional gestational hypertension cases were identified by reviewing blood pressure monitoring data from the EMR where systolic blood pressure ≥140 mmHg or diastolic blood pressure ≥90 mmHg was observed on at least two consecutive prenatal visits after 20 gestational weeks. Mothers with chronic hypertension (*N* = 15) without developing preeclampsia or eclampsia were excluded from the analyses of effect modification by HDP.

We examined prenatal distress as a secondary modifier, in order to test our hypothesis that the biological condition of HDPs, rather than any associated psychological stress, increased susceptibility to traffic-related air pollution. Prenatal distress was evaluated by the validated Prenatal Distress Questionnaire (PDQ) [46] administered at the third trimester study visit. The PDQ includes 17 items that assess both issues arising from early pregnancy and issues that become more relevant as pregnancy progresses. The questionnaire asked whether participants felt bothered, upset, or worried about “the effect of ongoing health problems such as high blood pressure or diabetes”, “feeling tired and having low energy during your pregnancy”, or “whether the baby might come too early”, etc. Responses were on a 3-point scale ranging from 0 (not at all) to 2 (very much). The total score was calculated by summing the responses to each item. Scores range from 0 to 34, with higher scores indicating greater prenatal distress. Higher prenatal distress was categorized as greater than or equal to the median PDQ score (median score = 7).

### Statistical analyses

Distributions of participant characteristics were summarized using means and standard deviations for continuous variables and frequencies and percentages for categorical variables. In all models, PDM scores were log-transformed due to their right skewed distribution. We first conducted multivariable linear regression models to assess the main effects of traffic-related NO_x_ on the log PDM scores evaluated at each postpartum timepoint separately, adjusted for covariates. We also evaluated effect modification by HDP status and PDQ score by adding the cross-product term into the linear regression models with the main effect of exposure. The interaction model was used to derive group-specific NO_x_ effect estimates. We explored possible non-linearity of traffic-related NO_x_ effects using generalized additive models (GAM) with a penalized spline for estimates of traffic-related NO_x_ for each road type separately, adjusting for the same covariates as the main models. No deviation of linearity of NO_x_ by any road class was detected for the main effect models. Collinearity was assessed by variance inflation factor (VIF) for each variable in the model.

We then performed multivariate mixed effect models including outcomes at 4 timepoints simultaneously with a random intercept for each mother and added the timepoint of outcome assessment (1, 3, 6, 12 months postpartum) as a covariate in the model, in order to evaluate the association across all timepoints in the first postpartum year for each participant. Since our data visualization using spaghetti plots of log(PDM) on time for each participant with an overlaid loess smoother (Fig. S5) indicated a non-linear relationship between time of assessment and PDM, the categorical month was applied to the models instead of the linear month. We also assessed whether the association of interest differed by timepoints of PDM assessment by adding the interaction terms of time of assessment * NO_x_ into the linear mixed effect models. The final linear mixed effect model did not include the interactions terms for parsimony given the interaction terms were non-significant (*p* > 0.05).

We conducted the following sensitivity analyses for all models to evaluate the robustness of our results: (1) exclusion of mothers with previous history of depression or antidepressant medication use; (2) exclusion of mothers who delivered preterm births; (3) additional adjustment of annual household income levels. For mixed effects models, we additionally performed the following sensitivity analyses to examine if the results were sensitive to participation at different timepoints: (1) removal of one PDM outcome assessment timepoint (out of four) in the models; (2) restricting to mothers who completed all four PDM assessments.

All analyses were based on two-sided alternative hypotheses with an alpha level of 0.05. Data linkages of different MADRES datasets were conducted using SAS Version 9.4, and other data management and analyses were performed in R Version 4.2.1.

## Results

### Descriptive statistics

Study population characteristics for the 453 participants are presented in Table 1. On average, maternal age was 28.47 years (SD = 5.95) at consent, and pre-pregnancy BMI was 29.09 kg/m^2^ (SD = 6.57). The majority of participants were Hispanic or Latina (84.1%). Participants had relatively low education level with about 29% not having completed high school. About 11% had a prior diagnosis of depression or antidepressant medication use. The sample demographic characteristics were similar to the demographic characteristics in the complete MADRES cohort [23].

**Table 1:** Participants characteristics of the study population (*N* = 453).

Variables	*N* (%) / Mean ± SD
Maternal age, years	28.47 ± 5.95
Maternal race and ethnicity	
Hispanic or Latina	381 (84.1%)
White, non-Hispanic	12 (2.6%)
Black, non-Hispanic	54 (11.9%)
Other, non-Hispanic	6 (1.3%)
Maternal education	
Less than high school	131 (28.9%)
High school graduate	144 (31.8%)
Some college or technical school	125 (27.6%)
Completed 4 years of college	46 (10.2%)
Some graduate training after college	7 (1.5%)
Annual household income	
Less than $30,000	220 (48.6%)
$30,000 or more	77 (17.0%)
Not know	156 (34.4%)
Pre-pregnancy BMI, kg/m^2^	29.09 ± 6.57
Pre-pregnancy depression history	
Yes in medical records	48 (10.6%)
Not recorded	405 (89.4%)
Parity	
0	141 (31.1%)
1	153 (33.8%)
2 or more	159 (35.1%)
Preterm birth	41 (9.1%)
Season of child’s birth	
Fall	118 (26.0%)
Spring	108 (23.8%)
Summer	96 (21.2%)
Winter	131 (28.9%)
Year of child’s birth	
2020	83 (18.3%)
2019	144 (31.8%)
2018	132 (29.1%)
2017	86 (19.0%)
2016	8 (1.8%)
Traffic-related NO_x_ across pregnancy, ppb	
Freeways/ Highways	1.77 ± 1.51
Major roads	0.30 ± 0.29
Minor roads	1.56 ± 0.72
Total	3.63 ± 1.71

*BMI* body mass index.

**Fig. 1:**
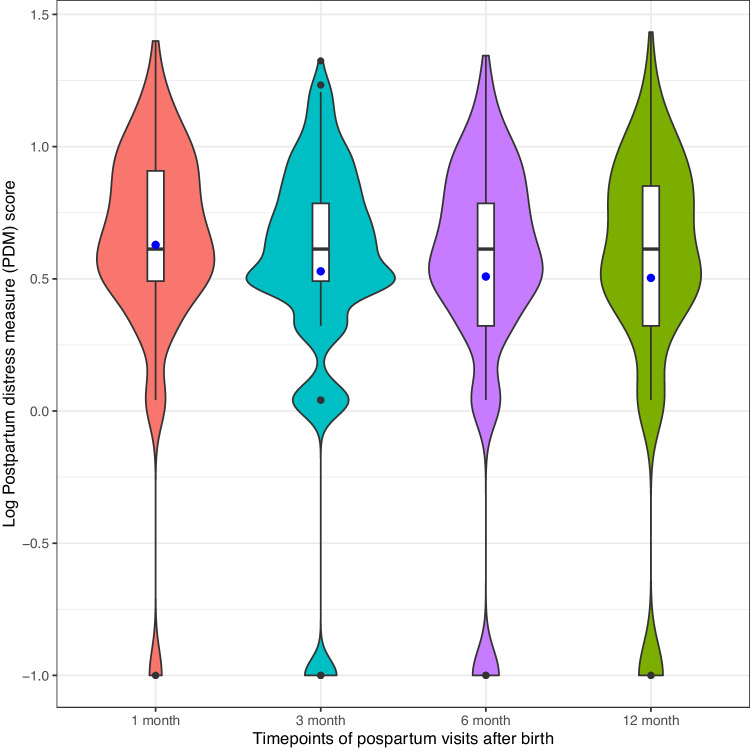
The distribution of log (PDM) scores at 1 month, 3 months, 6 months, and 12 months postpartum with violin plots and boxplots. The mean of log(PDM) scores at each time point are shown as blue dots.

The NO_x_ concentrations from freeways and highways were highest among three road classes, followed by NO_x_ levels from minor roads, and NO_x_ levels from major roads (Table 1). The distributions of log(PDM) scores at each postpartum timepoint are shown in Fig. 1. Average log(PDM) scores were fairly consistent at 3, 6, and 12 months and higher at 1 month postpartum. Log(PDM) scores at different months postpartum were significantly and moderately correlated with each other (Fig. S6).

### Overall and subgroup-specific association

**Fig. 2:**
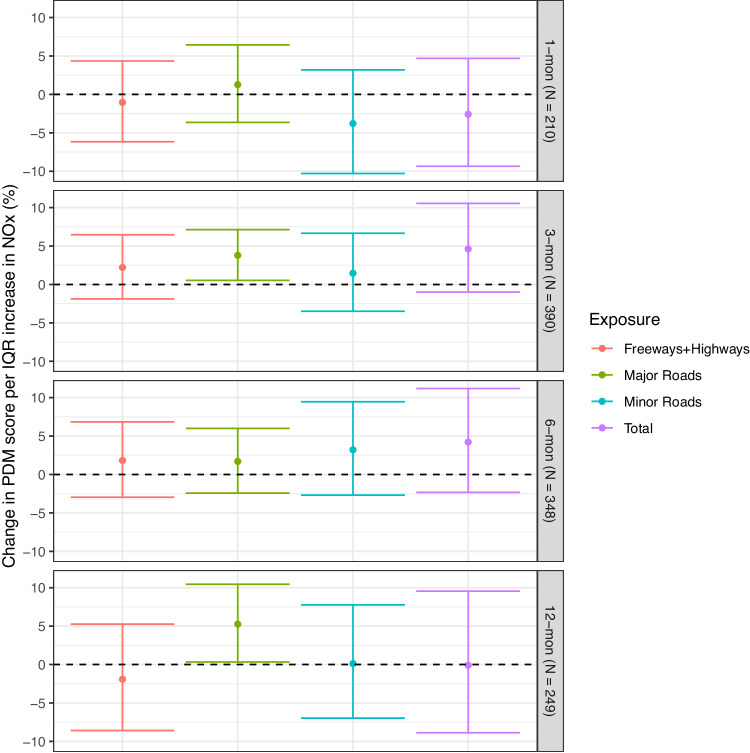
Estimated percent changes in 1-month, 3-month, 6-month, and 12-month postpartum distress measure (PDM) scores per IQR increase in total traffic-related NO_x_ and traffic-related NO_x_ for each road class during pregnancy based on multivariate linear regression (*N* = 453). *The models were adjusted for covariates including, maternal age, race/ethnicity, education, pre-pregnancy BMI, parity, season of childbirth, and calendar year of childbirth.

Figure 2 presents the percent change in the PDM score per interquartile range (IQR) increase in traffic-related NO_x_ for each road type after adjusting for covariates from timepoint-specific linear regression models. We found significant associations of prenatal traffic-related NO_x_ from major roads with PDM scores at 3 and 12 months postpartum. Specifically, we observed that women with an IQR increase in prenatal NO_x_ from major roads had 3.78% (95% CI: 0.53–7.14%) greater 3-month PDM scores, and 5.27% (95% CI: 0.33–10.45%) greater 12-month PDM scores. The association of prenatal traffic-related NO_x_ from major roads with PDM scores at 6 months was not statistically significant but showed a similar direction of effect. We observed a similar pattern of effects for total traffic-related NO_x_ on PDM scores at 3, and 6 months postpartum, although these associations did not reach statistical significance.

We observed non-significant positive associations between prenatal traffic-related NO_x_ from minor roads with PDM scores at 3, 6 and 12 months postpartum, and between prenatal traffic related NO_x_ from freeways and highways with PDM scores at 3 months and 6 months postpartum. Associations for prenatal traffic-related NO_x_ for all road classes with PDM scores at 1 month postpartum were not significant and showed no clear patterns.

**Fig. 3:**
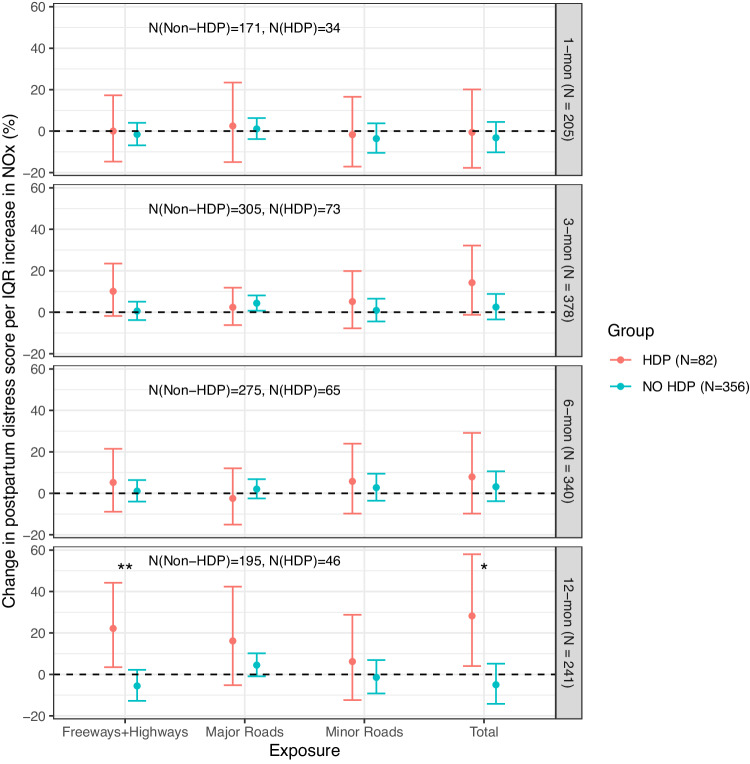
Estimated percent changes in 1-month, 3-month, 6-month, and 12-month postpartum distress measure (PDM) scores per IQR increase in total traffic-related NO_x_ and traffic-related NO_x_ for each road class during pregnancy by hypertensive disorders of pregnancy (HDP) status based on multivariate linear regression (*N* = 438). *The models were adjusted for covariates including, maternal age, race/ethnicity, education, pre-pregnancy BMI, parity, season of childbirth, and calendar year of childbirth. * marks indicate the p-value of effect modification by HDP status for the association of interest is less than 0.05. ** marks indicate the p-value of effect modification by HDP status for the association of interest is less than 0.01.

We also assessed effect modification by HDP diagnosis and PDQ scores. After excluding 15 participants with chronic hypertension, we identified 82 mothers with HDP and 356 mothers without any HDP. We observed that the presence of HDP significantly modified the association between prenatal traffic related NO_x_ from freeways and highways and total NO_x_ and 12-month PDM scores (Fig. 3). Specifically, among participants with any HDP, women with an IQR increase in traffic related NO_x_ from freeways and highways had 22.18% (95% CI: 3.49–44.23%, *p* for interaction < 0.01) greater 12-month PDM scores, compared to participants without HDP whose association was null (−5.55%; 95% CI: −12.73%, 2.21%). Similarly, among participants with HDP, an IQR increase in total traffic-related NO_x_ was associated with 28.22% (95% CI: 4.05–58.00%, *p* for interaction = 0.01) greater 12-month PDM scores, compared to participants without HDP whose association was also null (−5.00%; 95% CI: −14.20%, 5.20%). Similarly, we observed a stronger association between NO_x_ from minor roads and PDM scores at 12 months postpartum among mothers with HDP, in comparison to mothers without HDP (Fig. 3). However, the interaction analysis did not yield statistical significance. A similar pattern was also observed for 3-month and 6-month PDM scores (Fig. 3), although the interaction by HDP status was not statistically significant. We did not find any evidence for effect modification by prenatal distress symptoms on the association between prenatal traffic related NO_x_ and PDM scores (Fig. S7).

### Repeated outcome analyses: association between prenatal traffic-related NO_x_ and PDM scores across the first postpartum year

Table 2 displays the association between prenatal traffic-related NO_x_ for each road class and PDM scores across the first postpartum year. The association across the postpartum timepoints was consistent with the results based on the timepoint-specific linear regressions. Total traffic-related NO_x_ and NO_x_ from all road classes including freeways/highways, major and minor roads were all positively associated with PDM scores across the first postpartum year. For a given participant, an IQR increase in prenatal traffic-related NO_x_ from major roads was significantly associated with a 3.06% (95% CI: 0.43–5.76%) increase in PDM scores across the first year postpartum.

**Table 2:** Estimated percent change in postpartum distress measure (PDM) scores across the first year postpartum per IQR increase in total traffic-related NO_x_ and traffic-related NO_x_ for each road class during pregnancy from linear mixed effect models (*N* = 453).

NO_x_ (ppb)	% Change^a^	95% CI	*p*-value
Freeway/ Highway (1.32 ppb)	0.69	(−2.41, 3.90)	0.66
Major Road (0.22 ppb)	3.06	(0.43, 5.76)	0.02
Minor Road (0.81 ppb)	0.75	(−3.11, 4.77)	0.71
Total (2.02 ppb)	2.09	(−2.18, 6.54)	0.34

^a^The associations were estimated based on linear mixed effect models with a categorical time variable of the timepoints of outcome assessment (1 month postpartum, 3 months postpartum, 6 months postpartum, 12 months postpartum), adjusting for other covariates (maternal age, race/ethnicity, education, pre-pregnancy BMI, parity, season of childbirth, and calendar year of childbirth).

### Sensitivity analyses

The results of our study were robust to multiple sensitivity analyses. The pattern of the association based on linear regression models for the time-specific association did not change substantially after excluding mothers with a previous history of depression or antidepressant medication use (Fig. S8), after excluding mothers who delivered preterm births (Fig. S9), or after additionally adjusting for annual household levels in the model (Fig. S10). Also, we did not observe appreciable changes in the magnitude of the repeated measures association (from the mixed effect model) with total traffic-related NO_x_ and NO_x_ from different classes of roads NO_x_, after excluding mothers with a previous history of depression or antidepressant medication use (Table S2), after excluding mothers who delivered preterm births (Table S3), or after additionally adjusting for annual household income levels in the model (Table S4). The overall pattern of association persisted when “leaving out one” PDM assessment timepoint (out of four PDM timepoints) each time in the mixed effect models (Table S5). When we restricted to mothers who completed all four PDM assessments, the associations with NO_x_ from the larger road classes (freeway/highway, major) and total NO_x_ were larger compared to the primary models (Table S6). Also, the effect estimate for NO_x_ from major roads was larger but not statistically significant (*p* = 0.15) likely due to decreased sample size (Table S6).

## Discussion

In the predominately low-income Hispanic/Latina population that comprises the MADRES cohort, we found that prenatal traffic-related NO_x_ was positively associated with increased depressive and anxiety symptoms across the first year postpartum. We particularly observed increased symptoms of postpartum anxiety and depression associated with traffic-related NO_x_ from major roads. In addition, we found novel evidence for increased susceptibility to postpartum mental health effects from traffic-related NO_x_ among women with hypertensive disorders of pregnancy. Women with HDPs and higher exposures to prenatal traffic-related NO_x_ reported higher symptoms of postpartum depressive and anxiety symptoms.

To our knowledge, this is the first study to examine effects of TRAP from different roadway types on postpartum depressive and anxiety symptoms. We previously showed that ambient NO_2_ was associated with increased risk of depression at 12 months after childbirth [23]. Another prior study in Taiwan also demonstrated a significant association between NO_2_ (estimated based on a hybrid kriging/land-use regression (LUR) approach) during early pregnancy and PPD at 6 months postpartum [25]. Nevertheless, these models did not differentiate between the pollutants emitted near roadways and those originating from primary traffic pollution on a larger spatial scale, and did not account for oxides of nitrogen coming from sources other than traffic [26]. In contrast, our CALINE4 modeling approach exclusively estimated exposure to the mixture of vehicle emissions emitted near roadways.

We only found solid evidence of the association between NO_x_ from major roads and postpartum depressive and anxiety symptoms, although positive associations with NO_x_ from different road classes were observed. Possible reasons could be that major roads were closer to people’s residence compared to freeways and highways, indicating people have more chance of being exposed to fresh ultrafine particles, coarse particulate matter (PM_2.5-10_) exposure, and other gaseous components emitted from the near-roadway traffic [47]. Additionally, differences in the composition of pollutants from high-speed freeway vehicles, in contrast to the stop-and-go traffic patterns on major roads, could also explain the different association with NO_x_ from different road classes [26]. Notably, non-tailpipe emissions, such as coarse PM with trace metals [48], generated during stop-and-go traffic on major roads exhibit more pronounced toxicity in cellular systems compared to the PM generated from high-speed freeway vehicles [49]. Therefore, the effects of major roads might capture the effects of non-tailpipe, or other effects of traffic emission that are greater with proximity, whereas the effect of freeways+highways could capture more of the effects of tailpipe combustion emissions.

Our results are consistent with the limited studies that have found effects of ambient air pollution on PPD. For example, particulate matter exposure during pregnancy was positively associated symptoms of anhedonia and depression at 6 months postpartum in women in Mexico City [24]. Increased mid-pregnancy PM_2.5_ exposure was also found to be associated with increased depressive and anhedonia symptoms at 6 or 12 months after childbirth in primarily Hispanic and Black women [5]. Both studies used a widely accepted screening tool for PPD, the Edinburgh Postnatal Depression Scale (EPDS). The EPDS does not evaluate symptoms of anxiety, whereas the measure used in our study evaluates symptoms of depression, anxiety and obsessive-compulsive behaviors in the postpartum period [45].

Our findings indicated positive associations between prenatal traffic-related NO_x_ and postpartum depressive and anxiety symptoms at 3 months, and 6 months, and 12 months postpartum, whereas no suggestive pattern was found at 1 month postpartum. This may be supported by the difference in underlying mechanisms between postpartum blues versus PPD occurring at different times during thereafter. Postpartum blues—also called baby blues—are characterized by mood swings, irritability, and tearfulness, and are often reported to begin 2 to 3 days after delivery until up to 2 weeks [50]. Postpartum blues have been shown to be more related to deregulation of brain responses to the dramatic hormonal fluctuation during the peripartum period [51], whereas the underlying mechanism of long-lasting PPD involves a more complex combination of hormonal, genetic, environmental, and psychosocial factors [29]. In contrast, the pattern of association was more consistent across the other following three visits for outcome assessment, since the mechanisms underlying the depressive and anxiety symptoms may be more similar between later postpartum time points as compared to those at 1-month postpartum.

Our findings also suggest the combination of higher exposure to traffic-related NO_x_ and experiencing pregnancy complications such as HDPs may increase the risk of postpartum mood disorders. We did not find that prenatal distress symptoms conferred a similar vulnerability to the adverse effects of traffic pollution on postpartum distress symptoms despite a large body of literature documenting psychosocial stressors during pregnancy (e.g., prenatal depression, stressful life events during pregnancy, and poor social support) as independent risk factors for postpartum depression [27, 29, 50]. Studies have shown that HDP, especially preeclampsia, are associated with elevated levels of inflammatory markers such as C-reactive protein (CRP), tumor necrosis factor-alpha (TNF-alpha), and interleukin-6 (IL-6) [32, 33], and the inflammatory response in gestational hypertension is suggested to be associated with oxidative stress and endothelial dysfunction [37]. Air pollution’s effect on postpartum mental disorders may be mediated by inflammation and oxidative stress [18–21], suggesting shared mechanisms with HDPs. Other biological factors may also play a role in modifying the association between TRAP and postpartum mental health outcomes. Stress hormones, in particular those of the hypothalamic-pituitary-adrenal (HPA) axis, have been implicated in nonpuerperal depression [52, 53], and dysregulation of HPA axis was one of the proposed mechanisms of how air pollution influenced the mental disorders [54]. Our results showed increased susceptibility to traffic-related pollution effects in participants with HDP, but we found no evidence of increased vulnerability from prenatal stress, suggesting that future studies should investigate inflammatory pathways as potential mediators of these effects.

Our study has several strengths. First, its prospective design ensured a clear temporal relationship between exposure and outcome assessment, and the multiple visits during the postpartum period allow us to assess symptoms of anxiety and depression over time within a longitudinal modeling framework. Second, we also were able to leverage highly spatiotemporally resolved exposure models and daily residential histories that captured all participant mobility. Third, we aimed to address health disparities by conducting the study in a susceptible population of lower socioeconomic status, limited healthcare utilization for mental health care, and high environmental exposure levels. Fourth, the PDM was specifically developed to examine symptoms of both postpartum depression and anxiety which is an improvement over commonly used screening tools focusing on symptoms of postpartum depression alone. With a wider scope of symptomatology, the PDM could increase the likelihood of identifying women experiencing clinically significant levels of PPD, generalized anxiety, as well as obsessive compulsive disorder (OCD). The PDM was also designed to improve comprehension of items for U.S. women who may otherwise have difficulty understanding terminology in commonly used scales written by British researchers.

Inevitably, there were also limitations to our study. First, the local traffic-related NO_x_ exposure distribution in Los Angeles is different from other regions, making it hard to generalize our results to populations in other regions. Nevertheless, the results based on CALINE4 local NO_x_ estimates, the surrogate for the TRAP, could still provide important implications for urban planning, residence recommendation, and clinical intervention in the local setting. Second, CALINE4 is primarily a physical dispersion model, and it does not account for chemistry or secondary transformations. However, CALINE4 estimated the fresh vehicle emission signal from local near-roadway traffic without the further chemical processes over time. Third, we were unable to disentangle the effect of TRAP with pregnancy-wide road traffic noise, which has been shown to be a risk factor of depression after childbirth. The CALINE4 model captures near-roadway traffic emissions, meteorology, and road geometry, but cannot differentiate its correlation with traffic noise, however, CALINE4 NO_x_ estimates are highly correlated with the entire traffic emissions mixture including tailpipe and non-tailpipe emission, encompassing the potential latent traffic-related pollutants underlying the effect we assessed [48]. Future studies are warranted to explore the association between TRAP and postpartum depressive and anxiety symptoms after accounting for the role of the residential noise. Fourth, we were not able to capture the individual effects of other important pollutants from traffic such as carbon monoxide, PM, volatile organic compounds (VOCs), and polycyclic aromatic hydrocarbons (PAHs), despite that CALINE4 NO_x_ estimates are highly correlated with the entire traffic emissions mixture. Future research should encompass a comprehensive assessment of broader range of traffic-related pollutants. Fifth, potential bias may arise since we did not have an equal sample size for the four-time points of outcome assessment during the follow-up postpartum visits. There is a possibility that the participants’ underlying reasons for completing the PDM assessment at each time point were differential with respect to their risk of developing depressive and anxiety symptoms. However, the magnitude of the bias is expected to be minimal since the results remained stable after we removed one PDM assessment timepoint each time or after we restricted to participants with complete four timepoints PDM assessment in the mixed effect models. Furthermore, selection bias is a concern given there were eligible participants who delivered live births and reached 12 months postpartum by the cutoff day and did not complete the outcome assessment at any of the postpartum visit. However, we did not find evidence that women with higher prenatal depression were less likely to attend the postpartum visits within one year (Table S7), indicating that the selection bias attributed to differential participation may not be a major concern of the study. Additionally, while there is no established cutoff for the PDM suggesting clinically relevant depressive and anxiety symptoms that may confer greater risk of long-term health consequences, our findings regarding traffic-related air pollution’s adverse impacts on postpartum depressive and anxiety symptoms are highly relevant from a public health perspective given the ubiquity of the exposure and the high prevalence of postpartum mental health disorders that are associated with significant long-term health consequences. The results of our study identified traffic-related pollution as a potentially modifiable risk factor and showed a consistent pattern that pregnant people with higher traffic-related NO_x_ exposures may have increased symptoms of postpartum depression and anxiety. Lastly, while we did not find evidence of increased susceptibility to traffic-related NO_x_ among those participants who reported higher prenatal distress, further research is needed to examine the potential mediating role of prenatal stress, depression and anxiety in the relationship between of prenatal traffic-related NO_x_ and postpartum mental disorders. Similarly, underlying biological mechanisms including inflammatory pathways should be examined as potential mediators of this relationship.

## Conclusions

In this prospective cohort study, we found that prenatal local traffic-related NO_x_ was positively associated with increased postpartum depressive and anxiety symptoms across the first year postpartum among low-income Hispanic/Latina women residing in Los Angeles. We also demonstrated that women with HDP experienced stronger impacts of prenatal traffic-related NO_x_ on symptoms of postpartum depression and anxiety compared to women without HDPs, suggesting possible shared biological mechanisms. The findings suggest a need for urban planning strategies focusing on reducing TRAP, such as promoting alternative modes of transportation, implementing emission control measures, and creating green spaces. The results of this study also advance the understanding of the relationships between TRAP, maternal health during pregnancy and postpartum mental health, and have potential implications for clinical intervention to mitigate the effects of traffic-related pollution on postpartum mental health disorders.

## Supplementary information

Supplementary Information

## Data Availability

The datasets used and/or analyzed during the current study are available from the corresponding author on reasonable request and after approval by the USC Institutional Review Board. The codes used for the data analysis are available upon reasonable request for replication purposes.
