# Supplementary material for: Joint effects of traffic-related air pollution and hypertensive disorders of pregnancy on maternal postpartum depressive and anxiety symptoms
Source: J Expo Sci Environ Epidemiol. 2024 May 31;35(2):278–87. doi: 10.1038/s41370-024-00692-9 (PMC11607174; doi:10.1038/s41370-024-00692-9)
Supplement: Supplementary file 1 — Supplementary Information [file 41370_2024_692_MOESM1_ESM.docx]

**Supporting information**

**Joint Effects of Traffic-Related Air Pollution and Hypertensive Disorders of Pregnancy on Maternal Postpartum Depressive and Anxiety Symptoms**

**Content**

1. Table S1. The distribution of the average distance from participants’ residential addresses to roads of different classes during pregnancy
2. Table S2. Estimated percent changes in PDM scores across the first year postpartum per IQR increase in total traffic-related NO_x_ and traffic-related NO_x_ for each road class during pregnancy based on linear mixed effect models excluding pre-pregnancy depression history or antidepressant use (N = 405).
3. Table S3. Estimated percent changes in PDM score across the first year postpartum per IQR increase in total traffic-related NO_x_ and traffic-related NO_x_ for each road class during pregnancy based on linear mixed effect models excluding mothers who delivered preterm births (N = 412).
4. Table S4. Estimated percent changes in PDM scores across the first year postpartum per IQR increase in traffic-related NO_x_ for each road class during pregnancy based on linear mixed effect models after additionally adjusting for categorical annual household income (N = 453).
5. Table S5. Estimated percent changes in PDM scores across the first year postpartum per IQR increase in traffic-related NO_x_ for each road class during pregnancy based on linear mixed effect models leaving out one timepoint of PDM assessment each time.
6. Table S6. Estimated percent changes in PDM scores across the first year postpartum per IQR increase in traffic-related NO_x_ for each road class during pregnancy, restricting to participants who completed all four postpartum assessments (N = 119).
7. Table S7. The prevalence of depression (CES-D^a^ score ≥ 16) across pregnancy comparing participants in the final analytic population with participants who were eligible^b^ to complete postpartum assessments but did not participate in any postpartum assessment.
8. Figure S1. Consort diagram of total MADRES participants included in the final analysis
9. Figure S2. Spearman’s correlation between each trimester and across pregnancy for traffic-related NO_x_ from freeway/highway, major roads, minor roads, and total NO_x_ (all correlations had p-value < 0.05)
10. Figure S3. Spaghetti plots of PDM scores at each outcome assessment timepoint (months after childbirth) for each participant
11. Figure S4. Directed Acyclic Graphs (DAGs) between traffic-related NO_x_ and postpartum depressive and anxiety symptoms
12. Figure S5. Spaghetti plots of log(PDM) on timepoint of outcome assessment after childbirth for each participant with a overlaid loess smoother
13. Figure S6. Pearson’s correlation between log (PDM) scores at 4 time points (all the correlations had p-value < 0.05)
14. Figure S7. Estimated percent changes in 1-month, 3-month, 6-month, and 12-month postpartum distress measure (PDM) score per IQR increase in total traffic-related NO_x_ and traffic-related NO_x_ for each road class during pregnancy by prenatal distress questionnaire (PDQ) score (below and median score versus above median score) based on multivariate linear regression (N = 312). No interaction was significant (p-values < 0.05).
15. Figure S8. Estimated percent changes in 1-month, 3-month, 6-month, and 12-month postpartum distress measure (PDM) score per IQR increase in total traffic-related NO_x_ and traffic-related NO_x_ for each road class during pregnancy based on multivariate linear regression excluding participants with pre-pregnancy depression history or previous antidepressants use (N = 405).
16. Figure S9. Estimated percent changes in 1-month, 3-month, 6-month, and 12-month postpartum distress measure (PDM) score per IQR increase in total traffic-related NO_x_ and traffic-related NO_x_ for each road class during pregnancy based on multivariate linear regression excluding participants who delivered preterm birth (N = 412).
17. Figure S10. Estimated percent changes in 1-month, 3-month, 6-month, and 12-month postpartum distress measure (PDM) score per IQR increase in total traffic-related NO_x_ and traffic-related NO_x_ for each road class during pregnancy based on multivariate linear regression after additionally adjusting for annual household levels (N = 453).

**Table S1. The distribution of the average distance from participants’ residential addresses to roads of different classes during pregnancy.**

| Road class | Summary Statistics of the distance (m) | | | | | | |
| --- | --- | --- | --- | --- | --- | --- | --- |
|  | Mean | SD | Minimun | 25th percentile | Medium | 75th percentile | Maximum |
| Freeways+Highways | 1280.49 | 1279.74 | 68.52 | 466.55 | 993.08 | 1653.33 | 11963.50 |
| Major roads | 1063.30 | 2426.21 | 21.28 | 390.65 | 785.96 | 1290.27 | 48243.72 |
| Minor roads | 26.07 | 19.75 | 0.27 | 18.99 | 23.84 | 29.30 | 337.37 |

**Table S2. Estimated percent changes in PDM scores across the first year postpartum per IQR increase in total traffic-related NO_x_ and traffic-related NO_x_ for each road class during pregnancy based on linear mixed effect models excluding pre-pregnancy depression history or antidepressant use (N = 405).**

| **NO_x_ (ppb)** | **% Change^a^** | **95% CI** | **p-value** |
| --- | --- | --- | --- |
| **Freeway/ Highway** | 0.85 | (-2.44, 4.25) | 0.61 |
| **Major Road** | 2.94 | (-0.02, 5.99) | 0.05 |
| **Minor Road** | -0.56 | (-4.58, 3.63) | 0.79 |
| **Total** | 1.48 | (-2.97, 6.13) | 0.52 |

^a^ The associations were estimated based on linear mixed effect models with a categorical time variable of the timepoints of outcome assessment (1 month postpartum, 3 months postpartum, 6 months postpartum, 12 months postpartum). The models were also adjusted for other covariates (maternal age, race/ethnicity, education, pre-pregnancy BMI, parity, season of childbirth, and calendar year of childbirth).

**Table S3. Estimated percent changes in PDM scores across the first year postpartum per IQR increase in total traffic-related NO_x_ and traffic-related NO_x_ for each road class during pregnancy based on linear mixed effect models excluding mothers who delivered preterm births (N = 412)**

| **NO_x_ (ppb)** | **% Change^a^** | **95% CI** | **p-value** |
| --- | --- | --- | --- |
| **Freeway/ Highway** | 0.75 | (-2.50, 4.11) | 0.66 |
| **Major Road** | 3.30 | (0.27, 6.42) | 0.03 |
| **Minor Road** | 0.61 | (-3.51, 4.90) | 0.77 |
| **Total** | 1.95 | (-2.48, 6.59) | 0.39 |

^a^ The associations were estimated based on linear mixed effect models with a categorical time variable of the timepoints of outcome assessment (1 month postpartum, 3 months postpartum, 6 months postpartum, 12 months postpartum). The models also adjusted for other covariates (maternal age, race/ethnicity, education, pre-pregnancy BMI, parity, season of childbirth, and calendar year of childbirth).

**Table S4. Estimated percent changes in PDM scores across the first year postpartum per IQR increase in traffic-related NO_x_ for each road class during pregnancy based on linear mixed effect models after additionally adjusting for categorical annual household income (N = 453).**

| **NO_x_ (ppb)** | **% Change^a^** | **95% CI** | **p-value** |
| --- | --- | --- | --- |
| **Freeway/ Highway** | 0.49 | (-2.61, 3.70) | 0.76 |
| **Major Road** | 3.11 | (0.49, 5.81) | 0.02 |
| **Minor Road** | 0.75 | (-3.11, 4.77) | 0.71 |
| **Total** | 2.09 | (-2.18, 6.54) | 0.34 |

^a^ The associations were estimated based on linear mixed effect models with a categorical time variable of the timepoints of outcome assessment (1 month postpartum, 3 months postpartum, 6 months postpartum, 12 months postpartum). The models also adjusted for other covariates (maternal age, race/ethnicity, education, pre-pregnancy BMI, parity, season of childbirth, calendar year of childbirth, and annual household income levels).

**Table S5. Estimated percent changes in PDM scores across the first year postpartum per IQR increase in traffic-related NO_x_ for each road class during pregnancy based on linear mixed effect models leaving out one timepoint of PDM assessment each time.**

| **NO_x_** | **Excluding 1 month (N = 441)** | | |  | **Excluding 3 months (N = 413)** | | |  | **Excluding 6 months (N = 432)** | | |  | **Excluding 12 months (N = 448)** | | |
| --- | --- | --- | --- | --- | --- | --- | --- | --- | --- | --- | --- | --- | --- | --- | --- |
|  | **Estimate^a^** | **95%CI** | **p** |  | **Estimate^a^** | **95%CI** | **p** |  | **Estimate^a^** | **95%CI** | **p** |  | **Estimate^a^** | **95%CI** | **p** |
| **Freeway/ Highway** | 1.09 | (-2.25, 4.55) | 0.53 |  | 0.72 | (-2.90, 4.48) | 0.70 |  | 0.33 | (-3.05, 3.83) | 0.85 |  | 1.06 | (-2.09, 4.32) | 0.51 |
| **Major** | 3.40 | (0.63, 6.24) | 0.02 |  | 2.45 | (-0.69, 5.69) | 0.13 |  | 3.65 | (0.87, 6.50) | 0.01 |  | 2.64 | (-0.06, 5.40) | 0.06 |
| **Minor** | 1.65 | (-2.45, 5.93) | 0.44 |  | 1.16 | (-3.31, 5.84) | 0.62 |  | -0.42 | (-4.51, 3.85) | 0.84 |  | 0.73 | (-3.23, 4.84) | 0.72 |
| **Total** | 3.17 | (-1.43, 7.98) | 0.18 |  | 2.11 | (-2.85, 7.32) | 0.41 |  | 1.30 | (-3.30, 6.11) | 0.59 |  | 2.38 | (-1.95, 6.91) | 0.29 |

^a^ The estimates were interpreted as the percent changes in PDM score per IQR increase in traffic-related NO_x_ for each road class. All the associations were estimated based on linear mixed effect models with a categorical time variable of the timepoints of outcome assessment (1 month postpartum, 3 months postpartum, 6 months postpartum, 12 months postpartum), adjusting for other covariates (maternal age, race/ethnicity, education, pre-pregnancy BMI, parity, season of childbirth, and calendar year of childbirth).

**Table S6. Estimated percent changes in PDM scores across the first year postpartum per IQR increase in traffic-related NO_x_ for each road class during pregnancy, restricting to participants who completed all four postpartum assessments (N = 119).**

| **NO_x_ (ppb)** | **% Change^a^** | **95% CI** | **p-value** |
| --- | --- | --- | --- |
| **Freeway/ Highway** | 3.05 | (-4.62, 11.34) | 0.44 |
| **Major Road** | 4.33 | (-1.57, 10.58) | 0.15 |
| **Minor Road** | 1.99 | (-6.19, 10.89) | 0.64 |
| **Total** | 5.37 | (-4.21, 15.90) | 0.28 |

^a^ The associations were estimated based on linear mixed effect models with a categorical time variable of the timepoints of outcome assessment (1 month postpartum, 3 months postpartum, 6 months postpartum, 12 months postpartum). The models also adjusted for other covariates (maternal age, race/ethnicity, education, pre-pregnancy BMI, parity, season of childbirth, and calendar year of childbirth).

**Table S7. The prevalence of depression (CES-D^a^ score ≥ 16) across pregnancy comparing participants in the final analytic population with participants who were eligible^b^ to complete postpartum assessments but did not participate in any postpartum assessment.**

| Analytic population  (N = 453) | |  | Eligible population without any postpartum PDM assessment  (N = 165) | |
| --- | --- | --- | --- | --- |
| N | Prenatal depression prevalence (%) |  | N | Prenatal depression prevalence (%) |
| 452 | 33.41 |  | 81 | 30.86 |

**^a^** Center for Epidemiologic Studies-Depression Scale (CES-D)

^b^Participants who delivered live births and reached 12 months postpartum by August 2022

**
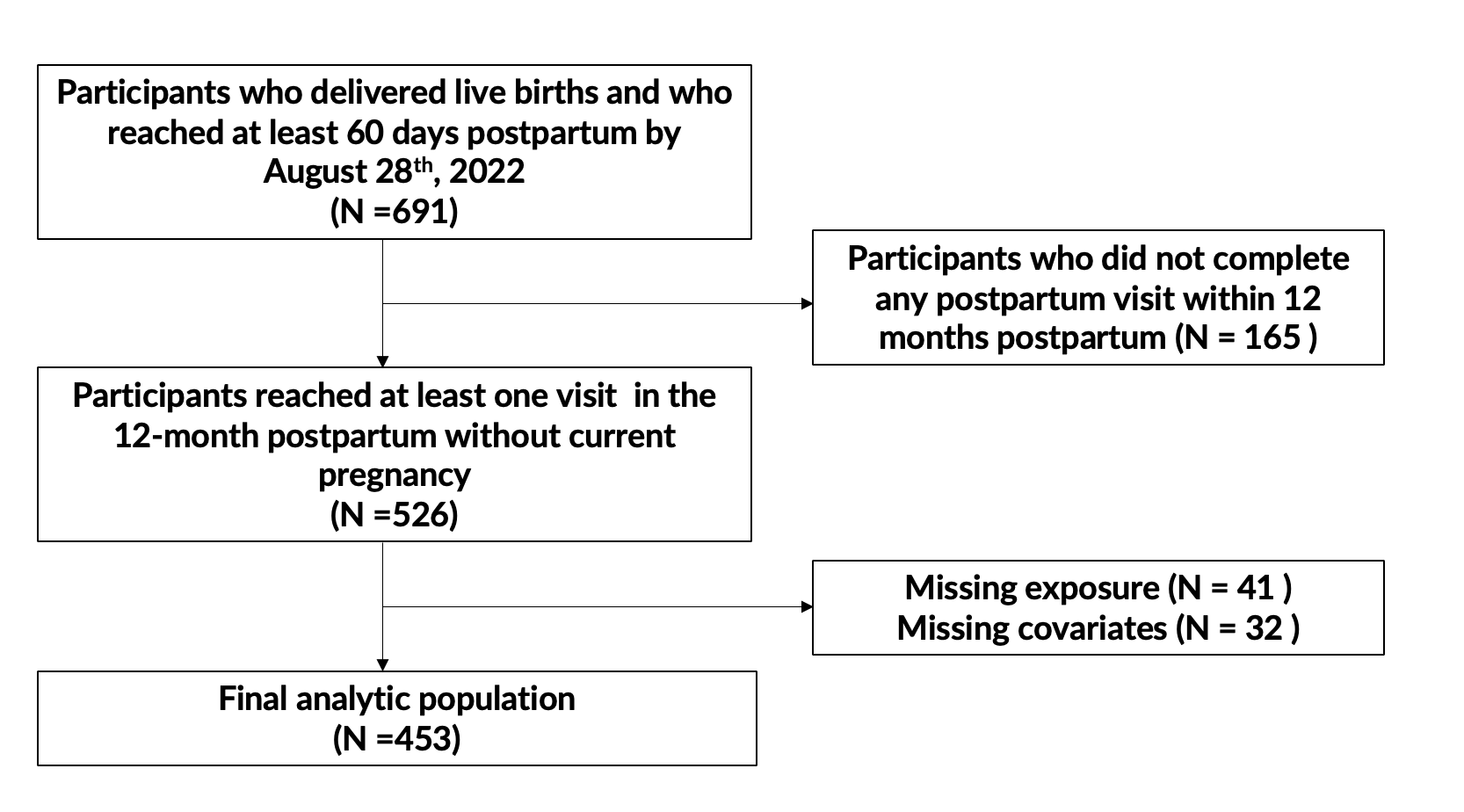
**

**Figure S1. Consort diagram of total MADRES participants included in the final analysis**

**
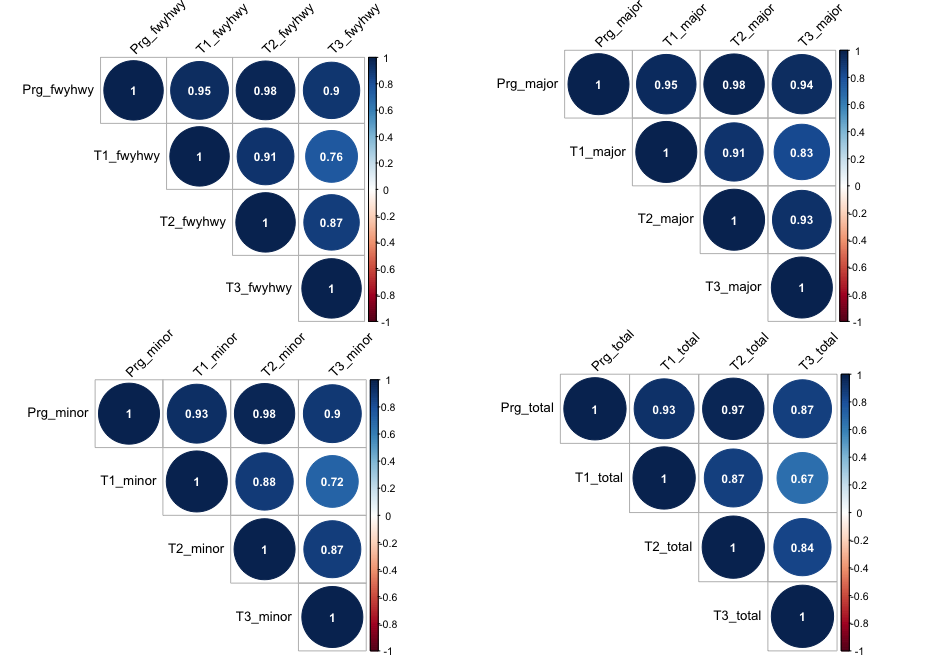
**

**Figure S2. Spearman’s correlation between each trimester and across pregnancy for traffic-related NO_x_ from freeway/highway, major roads, minor roads, and total NO_x_ (all correlations had p-value < 0.05)**

**
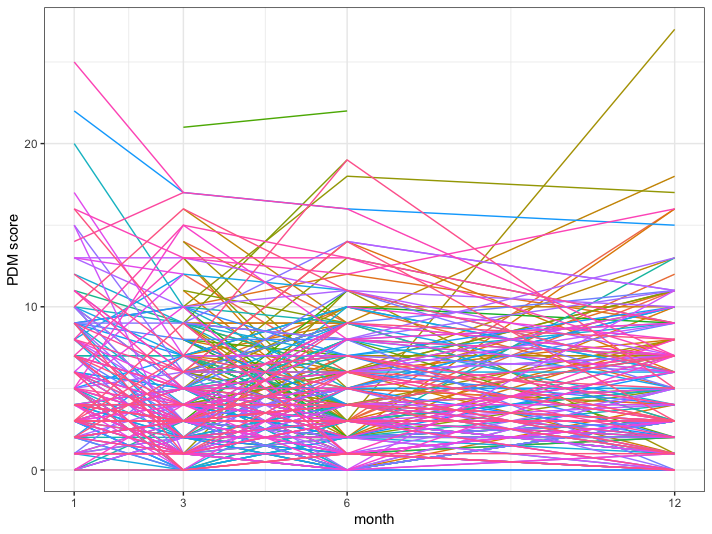
**

**Figure S3. Spaghetti plots of PDM scores at each outcome assessment timepoint (months after childbirth) for each participant**


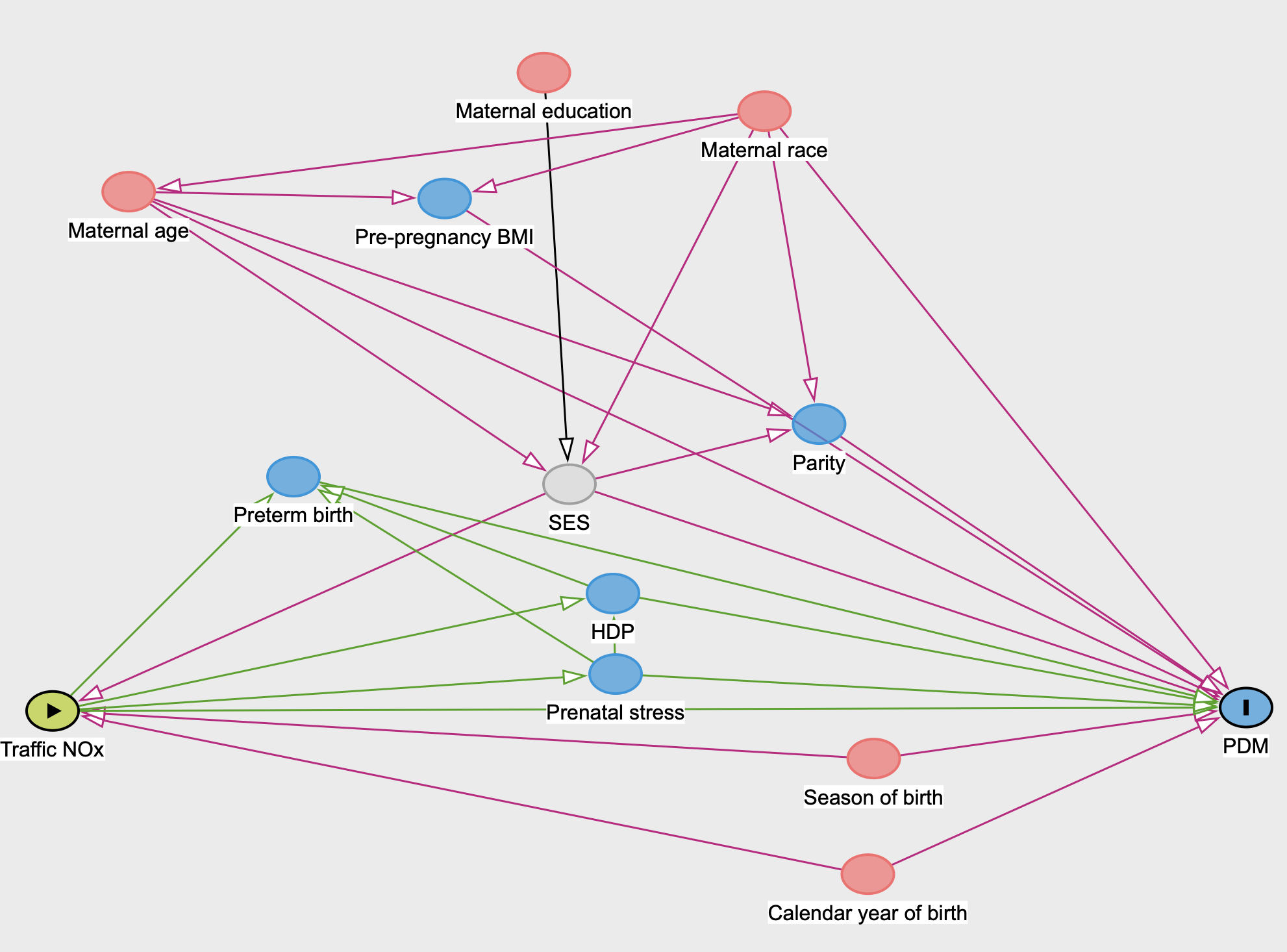


**Figure S4. Directed Acyclic Graphs (DAGs) between traffic-related NO_x_ and postpartum depressive and anxiety symptoms**

**Figure S5. Spaghetti plots of log(PDM) on timepoint of outcome assessment after childbirth for each participant with a overlaid loess smoother**


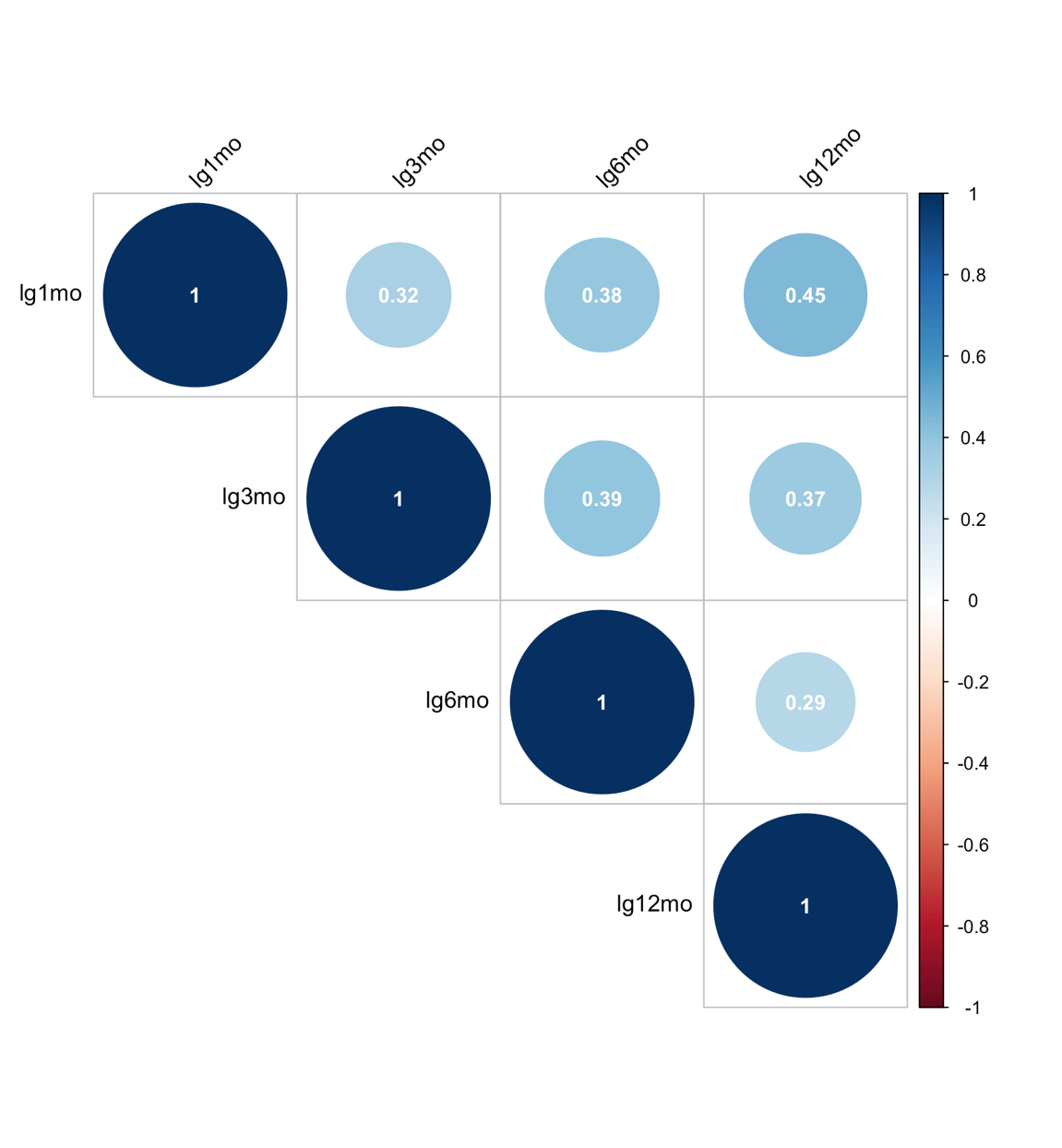


**Figure S6. Pearson’s correlation between log (PDM) scores at 4 timepoints (all the correlations had p-value < 0.05)**


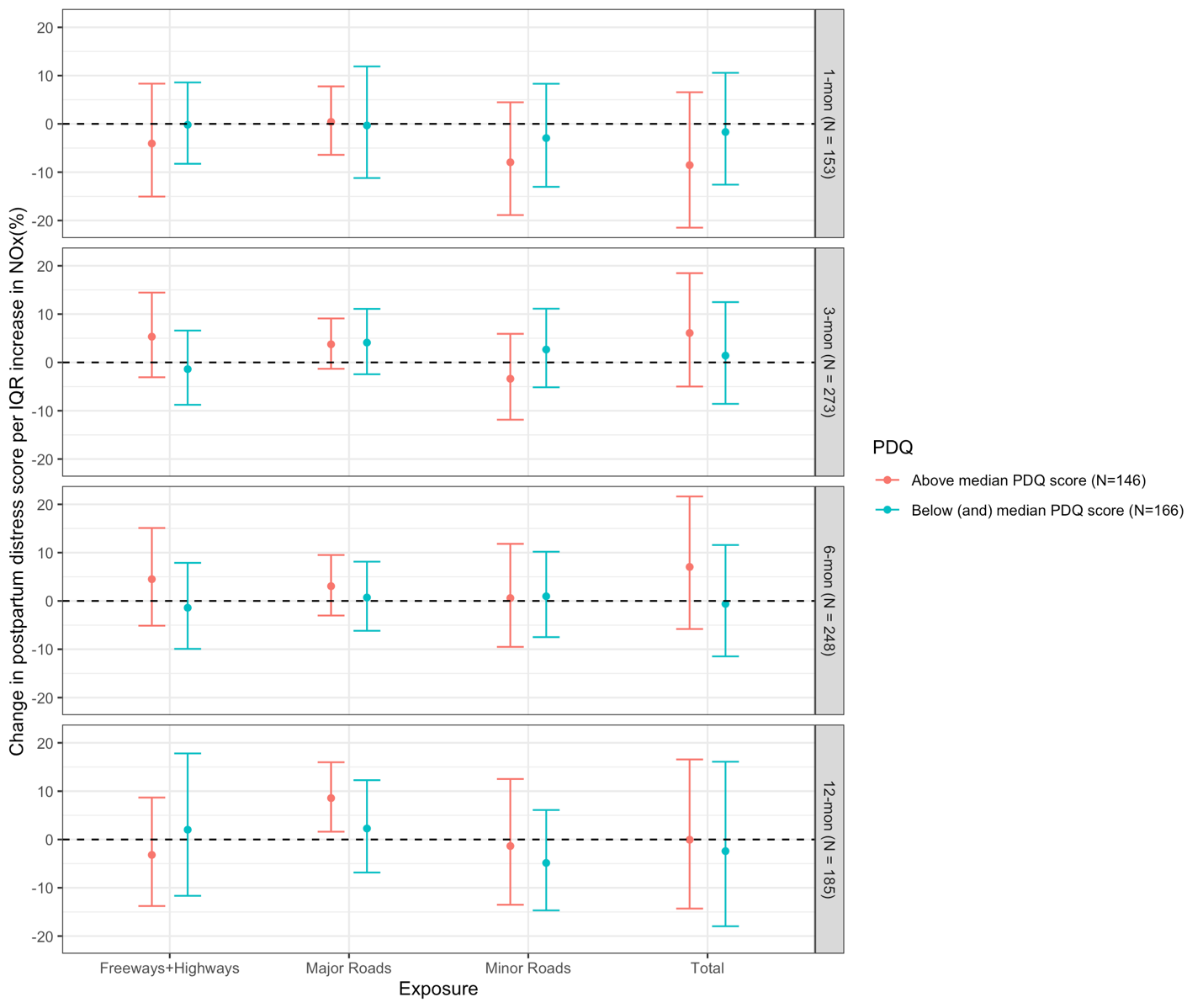


**Figure S7.** **Estimated percent changes in 1-month, 3-month, 6-month, and 12-month postpartum distress measure (PDM) score per IQR increase in total traffic-related NO_x_ and traffic-related NO_x_ for each road class during pregnancy by prenatal distress questionnaire (PDQ) score (below median score versus above median score) based on multivariate linear regression (N = 312). No interaction was significant (p-values < 0.05).**

*****The model adjusted for covariates including, maternal age, race/ethnicity, education, pre-pregnancy BMI, parity, season of childbirth, and calendar year of childbirth.

**Figure S8. Estimated percent changes in 1-month, 3-month, 6-month, and 12-month postpartum distress measure (PDM) score per IQR increase in total traffic-related NO_x_ and traffic-related NO_x_ for each road class during pregnancy based on multivariate linear regression excluding participants with pre-pregnancy depression history or previous antidepressants use (N = 405).**

*The model adjusted for covariates including, maternal age, race/ethnicity, education, pre-pregnancy BMI, parity, season of childbirth, and calendar year of childbirth.

**Figure S9. Estimated percent changes in 1-month, 3-month, 6-month, and 12-month postpartum distress measure (PDM) score per IQR increase in total traffic-related NO_x_ and traffic-related NO_x_ for each road class during pregnancy based on multivariate linear regression excluding participants who delivered preterm birth (N = 412).**

*The model adjusted for covariates including, maternal age, race/ethnicity, education, pre-pregnancy BMI, parity, season of childbirth, and calendar year of childbirth.

**Figure S10. Estimated percent changes in 1-month, 3-month, 6-month, and 12-month postpartum distress measure (PDM) score per IQR increase in total traffic-related NO_x_ and traffic-related NO_x_ for each road class during pregnancy based on multivariate linear regression after additionally adjusting for annual household levels (N = 453).**

*The model adjusted for covariates including, maternal age, race/ethnicity, education, pre-pregnancy BMI, parity, season of childbirth, calendar year of childbirth, and annual household income levels.
